# Silk scaffolding drives self-assembly of functional and mature human brain organoids

**DOI:** 10.3389/fcell.2022.1023279

**Published:** 2022-10-14

**Authors:** Edoardo Sozzi, Janko Kajtez, Andreas Bruzelius, Milan Finn Wesseler, Fredrik Nilsson, Marcella Birtele, Niels B. Larsen, Daniella Rylander Ottosson, Petter Storm, Malin Parmar, Alessandro Fiorenzano

**Affiliations:** ^1^ Department of Experimental Medical Science, Developmental and Regenerative Neurobiology, Wallenberg Neuroscience Center, Lund Stem Cell Center, Lund University, Lund, Sweden; ^2^ Department of Experimental Medical Science, Regenerative Neurophysiology, Wallenberg Neuroscience Center, Lund Stem Cell Center, Lund University, Lund, Sweden; ^3^ Department of Health Technology (DTU Health Tech), Technical University of Denmark, Kongens Lyngby, Denmark

**Keywords:** human pluripotent stem cells, cerebral organoid, silk scaffolding, tissue engineering, oxygen sensing

## Abstract

Human pluripotent stem cells (hPSCs) are intrinsically able to self-organize into cerebral organoids that mimic features of developing human brain tissue. These three-dimensional structures provide a unique opportunity to generate cytoarchitecture and cell-cell interactions reminiscent of human brain complexity in a dish. However, current *in vitro* brain organoid methodologies often result in intra-organoid variability, limiting their use in recapitulating later developmental stages as well as in disease modeling and drug discovery. In addition, cell stress and hypoxia resulting from long-term culture lead to incomplete maturation and cell death within the inner core. Here, we used a recombinant silk microfiber network as a scaffold to drive hPSCs to self-arrange into engineered cerebral organoids. Silk scaffolding promoted neuroectoderm formation and reduced heterogeneity of cellular organization within individual organoids. Bulk and single cell transcriptomics confirmed that silk cerebral organoids display more homogeneous and functionally mature neuronal properties than organoids grown in the absence of silk scaffold. Furthermore, oxygen sensing analysis showed that silk scaffolds create more favorable growth and differentiation conditions by facilitating the delivery of oxygen and nutrients. The silk scaffolding strategy appears to reduce intra-organoid variability and enhances self-organization into functionally mature human brain organoids.

## Introduction

The inaccessibility of human brain and the limited availability of human fetal tissue make studies on brain development, function and disease extremely challenging (Kelava and Lancaster, 2016; Di Lullo and Kriegstein, 2017; Fiorenzano et al., 2021c). In recent years, the emergence of brain organoids has brought about a major advancement in overcoming such issues. These three-dimensional (3D) tissue models have equipped researchers with a powerful *in vitro* platform that relies on the remarkable property of human pluripotent stem cells (hPSCs) to self-organize into complex 3D tissue structures that recapitulate architectural, functional, and transcriptional aspects of healthy and diseased human brain (Lancaster et al., 2013; Quadrato et al., 2017; Kanton et al., 2019; Bennett et al., 2021; Kelava et al., 2022). Current methodologies for the generation of human brain organoids can be broadly divided into two groups that follow either unguided or guided differentiation protocols (Arlotta and Pasca, 2019; Kim et al., 2020). The unguided approach leverages the intrinsic developmental potential of hPSCs and maximizes their inherent propensity to undergo spontaneous morphogenesis and self-driven differentiation. Consequently, the unguided route gives rise to brain organoids, commonly referred to as *cerebral* organoids, with complex structural patterning and cytoarchitecture akin to the native tissue of different brain regions. As such, these protocols provide an invaluable means to study cell-cell interactions and cellular diversity *in vitro* (Lancaster and Knoblich, 2014; Quadrato et al., 2017; Renner et al., 2017).

However, unguided protocols are susceptible to low reproducibility and unpredictable tissue heterogeneity both within and between individual organoids and batches. Brain organoids generated in this manner may also contain relatively large numbers of unwanted non-neuronal cells including derivatives of mesoderm and endoderm lineages. On the other hand, brain organoid protocols based on guided differentiation reduce cellular heterogeneity through the use of extrinsic patterning factors that induce hPSC toward the acquisition of cytoarchitectural identities mimicking individual brain regions such as dorsal and ventral forebrain, midbrain, and spinal cord (Bagley et al., 2017; Cederquist et al., 2019; Faustino Martins et al., 2020; Miura et al., 2020; Fiorenzano et al., 2021b). While guided brain organoid protocols provide an excellent platform to study region-specific features, the patterning factors used may reduce cell diversity and mask important developmental processes and refined phenotypes controlled by cell-cell interaction or finely controlled molecular gradients (Makrygianni and Chrousos, 2021; Nilsson et al., 2021; Sozzi et al., 2022). Furthermore, guided approaches do not fully address the limitations commonly encountered in the field of brain organoids such as inter-organoid variability, incomplete neuronal maturation, and the detrimental effects of insufficient tissue oxygenation leading to a hypoxic inner core (Pasca et al., 2019; Velasco et al., 2019; Bhaduri et al., 2020; Qian et al., 2020; Mansour et al., 2021). Therefore, there is an urgent need for novel solutions that are able to overcome these limitations while providing robust support for unguided hPSC differentiation.

To this end, bioengineering strategies aimed at generating homogeneous, mature, and healthy organoids are actively being pursued. Major efforts are being directed toward promoting more uniform tissue oxygenation as well as increased nutrient supply to and waste removal from cells throughout the organoid—tasks performed by the vascular network in native tissue. While ectopic vascularization in brain organoids has been achieved after transplantation into mouse cortex (Mansour et al., 2018), approaches to create vascular networks *in vitro* are still in the early phase of development and require advanced bioengineering systems (Cakir et al., 2019). As an alternative, researchers have resorted to cutting organoids mechanically into slice cultures (Qian et al., 2020) or have developed rotational mini-bioreactors and microfluidic chambers to enhance oxygen/nutrient diffusion *via* active flow of culture media leading to reduced hypoxia and enhanced functional maturation (Qian et al., 2016; Ao et al., 2020; Cho et al., 2021). It has also been hypothesized that biomaterial scaffolding (used in addition to the embedding hydrogel such as matrigel) could enhance cerebral organoid formation both structurally and functionally by templating hPSC aggregation, expansion, and differentiation along their backbones. To our knowledge, only two studies have tested this hypothesis (Lancaster et al., 2017; Rothenbucher et al., 2021). While these investigations confirmed that secondary biomaterial can direct hPSC self-organization into more homogeneous and reproducible neuroepithelial structures with enhanced gyrification by increasing the surface-to-volume ratio of the organoid (Lancaster et al., 2017; Rothenbucher et al., 2021), the use of synthetic scaffolding that is pseudo-3D at microscale, functionally inert, and without the ability to create microchannels limits the wider application of this approach (Lancaster et al., 2017; Rothenbucher et al., 2021).

Spider silk, a natural biomaterial with excellent biocompatibility and unique mechanical properties (Rising et al., 2011), holds great promise for unleashing the full potential of bioengineered scaffolding in organoid generation. Compared to silk fibroin, a protein obtained from *Bombyx mori* cocoons that has been extensively used in tissue engineering and shown to support 3D neuronal cultures (but not brain organoid formation) (Tang-Schomer et al., 2014; Sood et al., 2019; Rouleau et al., 2020), recombinant spider silk provides a highly reproducible and well-defined natural 3D scaffolding that is easy to handle and readily amenable (Johansson et al., 2019). We recently established that spider silk supports the successful generation of regionalized ventral midbrain organoids (Fiorenzano et al., 2021b). However, the effect of spider silk scaffolding on spontaneous morphogenesis and unguided hPSC self-patterning in cerebral organoids remains unexplored.

In this work, we showed that spider silk microfibers biofunctionalized with recombinant full-length human laminin assembled into a hierarchical 3D scaffold are able to instruct hPSCs to self-arrange into cerebral organoids. Importantly, the silk scaffolding assumes a delicate 3D structure at both the macro- and microscale that not only templates cellular seeding at the initial stages of organoid formation but also promotes the formation of interconnected microcavities as a form of passive vasculature. The presence of a silk scaffold enhanced neuroectoderm formation while reducing intra-organoid variability. Single cell transcriptomics revealed that a greater number of neurons are formed and that mature molecular features of human corticogenesis are better recapitulated within silk organoids cultured long term. This is furthermore accompanied by reduced activation of cellular stress pathways. Importantly, optical 3D mapping of tissue oxygenation revealed that the oxygen supply was increased in silk organoids, thus minimizing the formation of an inner necrotic core. Our silk scaffolding approach therefore provides a novel and reproducible solution for the generation of healthy cerebral organoids.

## Results

### Generation of silk cerebral organoids

**FIGURE 1:**
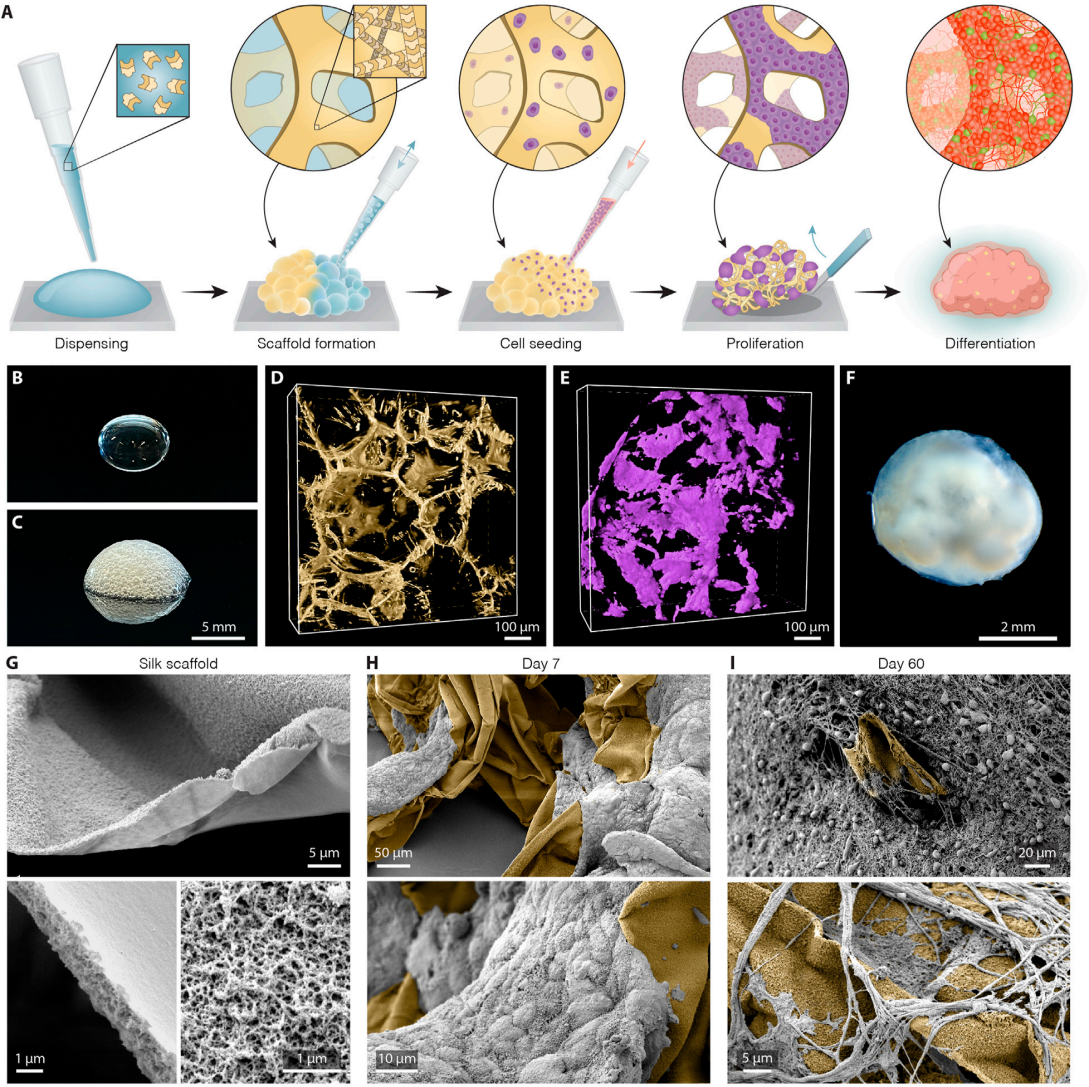
Generation of silk human cerebral organoids. **(A)** Schematic overview of silk scaffolding strategy for generation of silk human brain organoid. **(B,C)** Representative image of silk **(B)** in a liquid form as a droplet and **(C)** as a foam after introduction of air bubbles. Scale bar 5 mm. **(D)** Representative view of confocal fluorescence image stack of the polymerized silk scaffold (yellow) on day 5 of differentiation. Scale bar 100 µm. **(E)** Confocal image reconstructing in 3D live hPSCs (lilac) seeded along the silk fiber network. Scale bar 100 µm. **(F)** Representative bright-field image of long-term silk cerebral organoid culture (120 days). Scale bar 2 mm. **(G)** Scanning electron images (SEM) of silk scaffold without cells showing polymerized silk microfibers built up of nanofibrils. Scale bars 5 µm (top), 1 µm (bottom). **(H)** SEM images showing adherence and growth of hPSCs (grey) along the length of silk microfibers (yellow) at day 7. Scale bars 50 µm (top), 10 µm (bottom). **(I)** SEM images showing silk scaffold (yellow) with adherent neuronal cells and formation of intricate axon projections within silk engineered human cerebral organoids at day 60. Scale bars 20 µm (top), 5 µm (bottom).

To establish the bioengineered human brain organoid platform, we used a silk scaffold generated *via* a simple, easily adaptable, and reproducible approach (overall methodological concept presented in Figure 1A). The recombinant spider silk (Widhe et al., 2016) scaffold was biofunctionalized with full length human laminin 111, an important extracellular matrix protein that provides cell-adhesion motifs for stable neuronal adhesion, expansion, and maturation (Kirkeby et al., 2017; Fiorenzano et al., 2021a; Fiorenzano et al., 2021b). To form the scaffolding, air bubbles were introduced into droplets of ice-cold silk protein solution *via* repeated pipetting (Figures 1B,C). Restricted to thin layers between air pockets, soluble silk proteins polymerize at 37 °C into a 3D network of fibers formed around the wall of each air bubble, thereby producing a temporary foam in which undifferentiated hPSCs were dispersed. Attachment of cells to the scaffold is accompanied by the evacuation of air from the bubbles by diffusion into the surrounding cell culture media and by cellular consumption. This process results in a creation of a self-supported 3D network of fibers riddled with microcavities that successfully serves as a substrate for hPSC differentiation (Figure 1D). As can be seen by 3D fluorescence confocal microscopy, the cells propagated progressively along the surface of the formed scaffold and filled the inner space between silk fibers (Figure 1E). During this phase, the silk scaffold was attached to the bottom of an ultra-low attachment plate, generating a stationary engineered 3D culture. After 5 days, the culture was switched to neural induction media able to generate extensive cellular diversity, following a well-established and widely used protocol for the generation of cerebral organoids (Lancaster and Knoblich, 2014). Eight days after the start of differentiation, once the cells had filled the entire scaffold and the anchored organoid had grown in size, it was lifted from the bottom of the well to continue culturing in floating conditions, facilitating more efficient exchange of nutrients and oxygen (Figure 1F). Scanning electron microscope (SEM) imaging of the generated scaffold and subsequent organoid formation showed that silk, templated by air bubbles, polymerizes into flat microfibers with thickness of ∼1 µm and composed of defined nanofibrils (Figure 1G). Notably, nano-topography could play an important role by facilitating and enhancing cell adhesion and neuronal growth even on cell-inert substrates (Asif et al., 2020). SEM images further show that both neuronal progenitors interact directly with the silk surface and successfully attached along the length of the silk microfibers in the engineered organoids (Figure 1H) while differentiating into mature neurons with projections extending along the silk scaffold (Figure 1I, Supplementary Figure S1A,B).

Compared to cerebral organoids conventionally generated using the same protocol (termed “non-silk”) as a control, silk organoids displayed a less clear boundary between outer and inner regions, as well as the absence of a distinct core (Supplementary Figure S1C,D). Noteworthy, when the silk bubbles burst during silk organoid differentiation, they leave an empty space that will only be partially filled by cells (Figures 1G–I). This generates micropore structures that become smaller and smaller during stem cell differentiation (Supplementary Figure S1E,F).

### Silk scaffolding enhances neural induction in cerebral organoids

**FIGURE 2:**
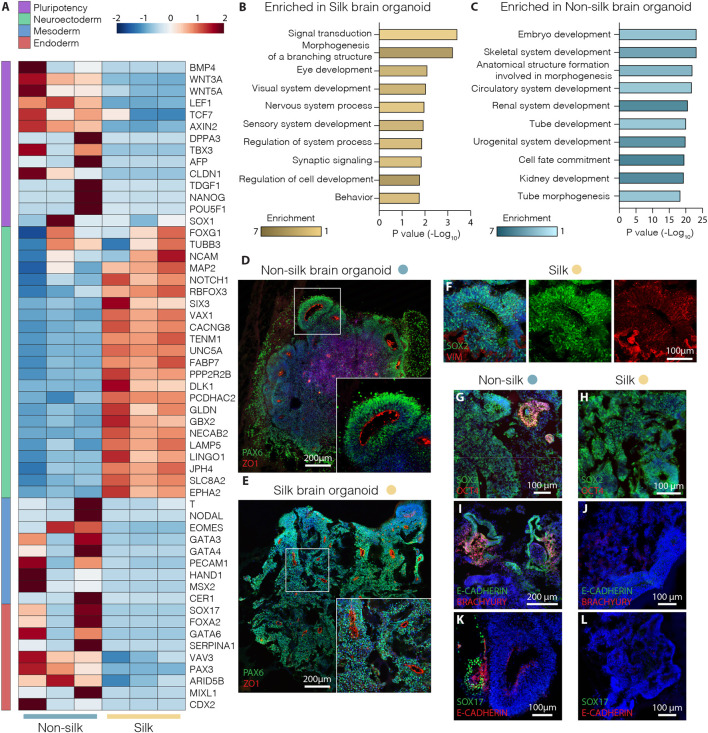
Silk cerebral organoids display enhanced neurogenesis. **(A)** Heat map showing expression levels of selected differentially expressed genes (DEG) considered markers of pluripotency and neuroectoderm, meso-endoderm differentiation in three biological replicates of silk and non-silk human brain organoids at day 20. **(B,C)** Enrichment of top ten biological process gene ontology terms ordered by *p*-value of upregulated genes in **(B)** silk and **(C)** non-silk brain organoids at day 20. **(D,E)** Immunohistochemistry of PAX6 and ZO1 showing *neural rosettes* in non-silk **(D)** and silk brain organoid **(E)** at day 20. Scale bars 200 µm. **(F)** Cryosection of silk brain organoid at day 20 showing SOX2/VIM double staining. Scale bar 100 µm. **(G,H)** Immunohistochemistry of SOX2/OCT4 in **(G)** non-silk and **(H)** silk organoid at day 20. Scale bars 100 µm. **(I–L)** Immunohistochemistry of **(I,J)** E-CAD/BRA and K,L) E-CAD/SOX17 in organoids grown with or without silk scaffold at day 20. Scale bars 200 µm **(I)** and 100 μm **(J–L)**. Nuclei were stained with DAPI.

Whole transcriptome expression analysis was performed using RNA-seq to directly compare silk and non-silk organoids at day 20 of differentiation, analyzing three biological replicates of each culture condition. We found a total of ∼900 differentially expressed protein-coding genes (≥2-fold, adjusted *p*-value < 0.05) in the two different organoid culture conditions (Supplementary Figure S1G). In comparison to cerebral organoids grown without silk microfibers, silk organoids showed more robust upregulation of neuroectodermal markers (*FABP7*, *GBX2*, *FOXG1*, *NCAM1*) and complete downregulation of pluripotency-associated genes (*DPPA3*, *NANOG*, *LEF1*, *CLDN1*) (Figure 2A). In contrast, a different set of genes was found uniquely upregulated in non-silk organoids (Figure 2A), indicating the presence of non-neural identities at an early stage of brain organoid differentiation as revealed by mesodermal- and endodermal-related genes (*NODAL, SOX17*, *FOXA2*, *GATA4*) (Figure 2A and Supplementary Figure S1G). These findings were further validated by quantitative PCR (qPCR) (Supplementary Figure S1H–J). Additionally, Gene Ontology analysis of brain organoids grown with silk microfibers revealed a significant enrichment of terms all related to neurodevelopment including *Nervous System,* and *Sensory* and *Synaptic pathways* (Figures 2B,C).

Consistent with RNAseq data, silk organoids established an internally uniform cell organization with the formation of radially organized cell structures resembling the neuroepithelial rosettes commonly described in cerebral organoids (Figures 2D,E and Supplementary Figure S1H). After 20 days of differentiation, intra-organoid variability was reduced in the silk organoids, shown by the emergence of homogeneous rosette-like structures expressing PAX6/SOX2/ZO1 (Figures 2D–F) throughout both the core and the edge. By contrast, non-silk organoids exhibited ectopic expression of BRA and SOX17 (Figure 2I–L and Supplementary Figure S1J) as well as retaining OCT4 levels in spatially restricted areas (Figures 2G,H and Supplementary Figure S1I). These findings show that the presence of silk scaffolds guides hPSCs to more precisely self-pattern toward neuroectodermal identity during cerebral organoid differentiation.

### scRNAseq reveals mature neuronal molecular features in silk-engineered cerebral organoids

**FIGURE 3:**
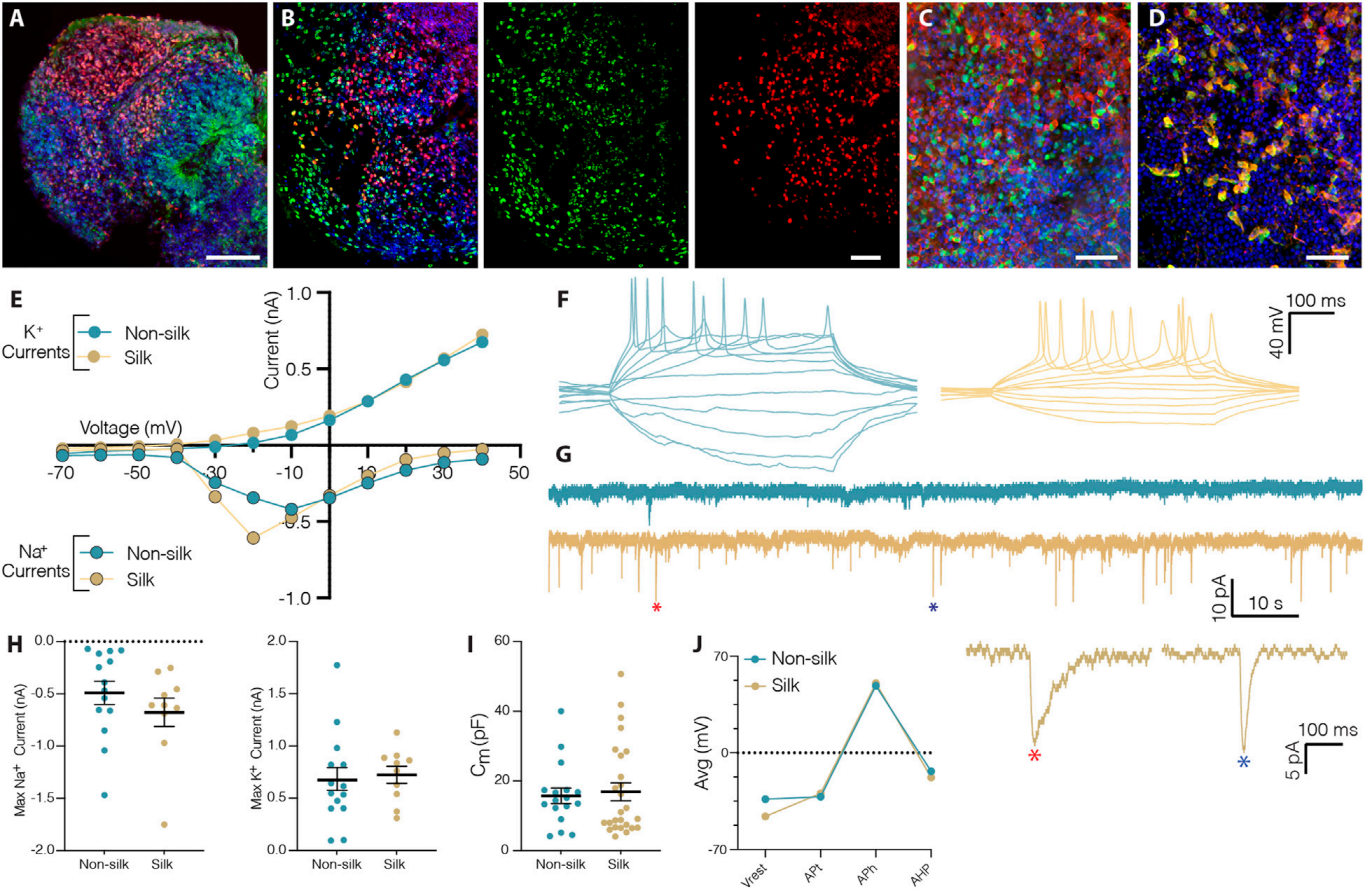
Silk scaffolding sustains functionally mature neurons generation. **(A)** Immunohistochemistry of FOXG1/CTIP2 showing cortical plate layer formation in silk brain organoid at day 60. Scale bar 100 µm. **(B)** Cryosection of silk brain organoid at day 120 showing SATB2/CTIP2 double staining. Scale bar 50 µm. **(C,D)** Immunohistochemistry of **(C)** VGLUT1/MAP2, **(D)** GABA/MAP2 showing distinct neuronal identities within silk brain organoids at day 120. Scale bars 50 µm. **(E)** Inward Na^+^ and outward K^+^ currents plotted as a function of stepwise voltage induction of cells in non-silk (n = 14) and silk (n = 10) organoids at 3 months. **(F)** Representative trace of evoked action potentials under rheobase current injection steps of cells in non-silk and silk organoids after 3 months in culture. **(G)** Sample traces of spontaneous activity recorded in voltage clamp, including magnification of selected postsynaptic events. **(H)** Quantification of maximum inward sodium current and maximum outward potassium current in silk (n = 14) and non-silk (n = 10) organoids after 3 months in culture. **(I)** Quantification of membrane capacitance in silk (n = 26) and non-silk (n = 17) organoids after 3 months in culture **(J)** Average value of action potential (AP) properties, resting membrane potential (V_rest_), AP threshold (AP_t_), AP amplitude (AP_h_), and afterhyperpolarization (AHP). n = 5 silk, n = 5 non-silk, 3 months. Nuclei were stained with DAPI.

Histological analysis revealed that silk cerebral organoids self-organized into different forebrain regions including those with ventral and dorsal forebrain cellular identities (Supplementary Figure S2A–D). As previously reported for conventional 3D cultures, silk brain organoids also presented a mature cortical layer organization (Figures 3A,B and Supplementary Figure S2E,F) (Lancaster et al., 2013; Quadrato et al., 2017). At month 2, cortical plate layer formation was defined by the presence of FOXG1^+^ and CTIP2^+^ neuronal cells enriched in the internal and external layer respectively (Figure 3A and Supplementary Figure S2E). At month 4, further segregation into early-born CTIP2^+^ neurons in the inner and late-born SATB2^+^ neurons in the outer layer defined a more mature degree of cortical organization (Figure 3B and Supplementary Figure S2F). The expression of glutamatergic (Vglut1) and GABAergic (GABA, GAD65/67) markers pointed to the generation of mature neuronal cells within silk cerebral organoids (Figures 3C,D and Supplementary Figure S2G–L). Patch-clamp electrophysiological recordings performed at 3 months confirmed the functional maturation of neurons (Figures 3E–J). As in non-silk organoids, voltage-gated sodium (Na^+^) and potassium (K^+^) currents indicated the active neuronal state within silk organoids (Figures 3E,H). In line with these findings, we also found that neurons in both silk and non-silk organoids were able to elicit current-induced action potentials (APs). However, while neurons in the conventional 3D culture fired single APs, neurons within silk brain organoids were able to fire multiple APs (Figure 3F) revealing a more mature neuronal profile. Examining the AP discharge pattern and cell-intrinsic properties, we found that threshold (APt), amplitude (APh), afterhyperpolarization (AHp) and resting membrane potential (Vrest) all remained similar across these two 3D culture conditions despite the difference in AP frequency (Figures 3I,J). We next evaluated the synaptic network by analyzing post-synaptic currents. While neurons in the non-silk cerebral organoids did not display any post-synaptic currents, multiple neurons (n = 7/20) in silk organoids exhibited post-synaptic currents indicative of a functional neuronal network, suggesting that silk 3D cultures facilitate formation of functional neuronal circuitries (Figure 3G).

**FIGURE 4:**
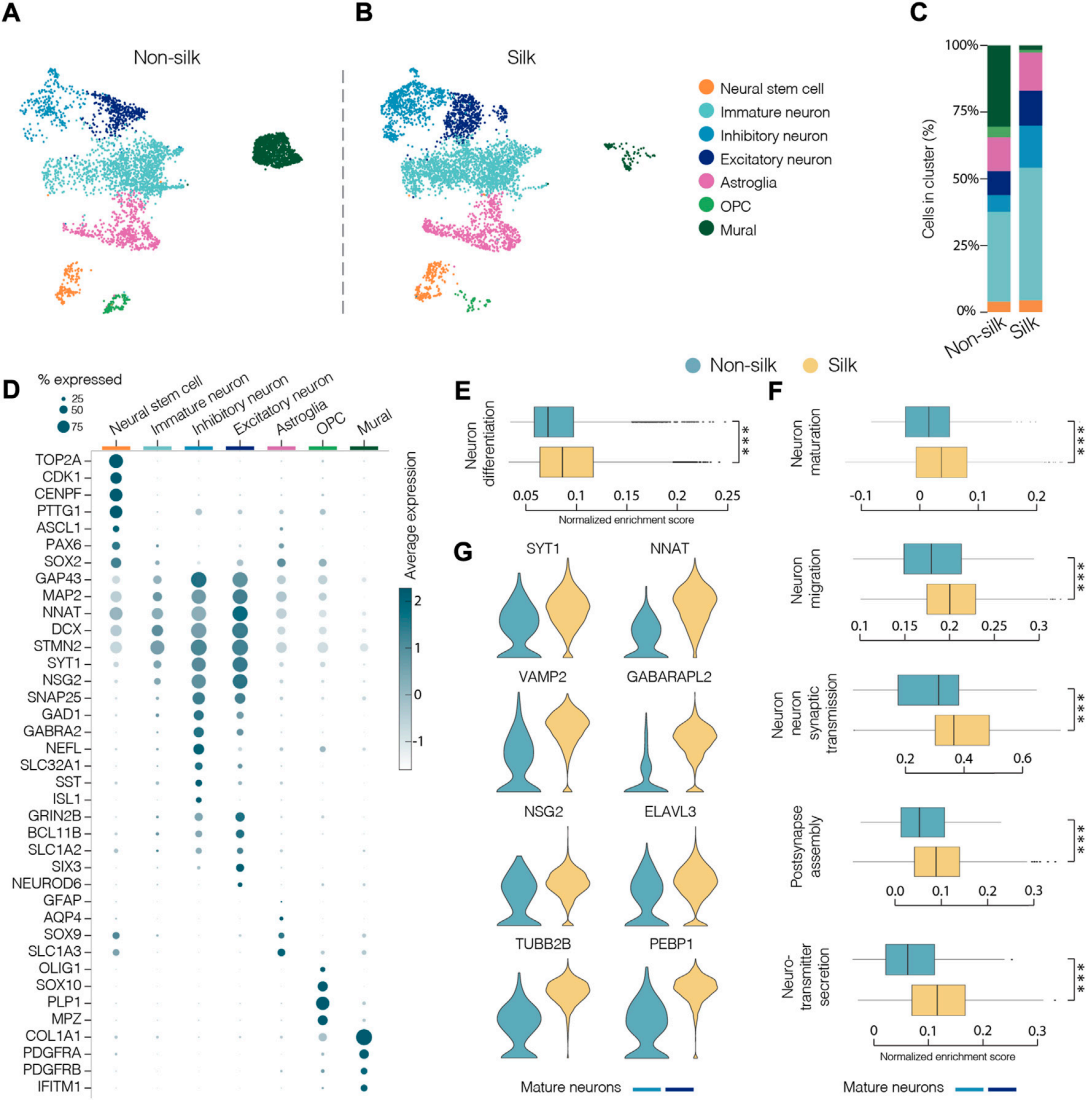
Single-cell transcriptomics reveals mature neuronal features in silk cerebral organoids. **(A and B)** 2D scatter plot of uniform manifold approximation and projection (UMAP) embeddings of non-silk H) and silk I) organoids at day 120 each showing clustering of ∼4,500 randomly selected cells. Colors indicate different cell type assignments. OPC, oligodendrocyte progenitor cells. **(C)** Bar plot showing the percentage of cells belonging to each cluster from silk and non-silk organoids. **(D)** Dot plot showing expression levels of indicated genes in each cluster. Indicated genes are established markers for neural stem cells, inhibitory and excitatory neuronal subtypes, astroglia, OPC, and mural cells. **(E)** Gene set enrichment analysis (GSEA) showing a statistically significant different score in *Neuron differentiation* term in non-silk and silk brain organoids. ***p < 0.001, two-tailed Wilcoxon rank sum test. **(F)** GSEA of *Neuron maturation, Neuron migration, Synaptic transmission, Post-synapse assembly and Neurotransmitter secretion* terms in mature neuronal clusters. ***p < 0.001, two-tailed Wilcoxon rank sum test. **(G)** Expression of representative mature neuronal markers in non-silk and silk brain organoid neuronal clusters after 4 months in culture.

To comprehensively characterize and compare cellular composition and neuronal identities within cerebral silk and non-silk organoids, we performed droplet-based single cell sequencing (10x) after 4 months in culture. Uniform manifold approximation and projection (UMAP) and graph-based clustering visualized seven major clusters after single cell data integration using Harmony (https://www.nature.com/articles/s41592-019-0619-0). We manually annotated the identified cell clusters by analyzing genes with highest differential expression, which were then used together with canonical cell type markers (Figures 4A,B). We also used previously published single cell datasets of cerebral organoids (Kanton et al., 2019) to confirm our cell type annotation by projecting the reference labels onto our data using Seurat’s v4 label transfer (Supplementary Figure S3A–C). UMAP revealed that similar cell types were formed in both non-silk and silk-bioengineered cerebral organoids (Figures 4A–C). After 4 months, a small cluster of *Neural stem cells* (orange) could still be detected in the UMAP space and was made up of cells expressing neural markers (*ASCL1*, *PAX6*, *SOX2*) with a proliferative signature (*TOP2A*, *CDK1*, *CENPF*) (Figures 4A,B,D and Supplementary Figure S3D,E). Cells in the turquoise cluster mainly differed from *Neural stem cells* in that they exited from cell cycle while retaining the expression of neural progenitor markers (*SOX2*, *PAX6*) (Figures 4A,B,D and Supplementary Figure S3D,E). We named this cluster *Immature neurons*, as these cells also acquired the expression of early neuronal markers (*STMN2*, *DCX*, *MAP2*) but lacked a clear mature neuronal signature (Figure 4D and Supplementary Figure S3E). A large fraction of cells was assigned to neuronal identity at this developmental stage, and they were enriched for markers of cerebral cortex neurons (*NEUROD6*, *BCL11b*, also known as *CTIP2*) (Figures 4A–C). Both excitatory (*SLC1A2*, *GRIN2B*, *SLC17A7* also known as *VGLUT1*) (dark blue) and inhibitory (*GAD1*, *GABRA2*, *SST*) (light blue) neuronal cell types emerged in both cerebral organoids grown with and without scaffolding (Figures 4A–D and Supplementary Figure S3E). Within the excitatory neuronal cluster, molecular features related to neural retina (*SIX3*, *GBN1*) were also detected (Supplementary Figure S3E). Alongside neuroectodermal-derived cell types, we were able to detect astroglia (*GFAP, AQP4, RFX4*) as well as a small group of oligodendrocyte progenitors (OPC, *OLIG1, OLIG2, SOX10*) (Figure 4D and Supplementary Figure S3E–G). We also detected a cluster of cells expressing markers of mural cells (*COL1A1, IFITM1, S100A11*) as well as genes involved in mesoderm commitment pathway (*FOXP1, FOXC1, CD248*) (Figures 4A–D and Supplementary Figure S3E,H–K) similar to Kanton et al. (Kanton et al., 2019). These mural cells are distinct from a newly identified class of cells termed VLMCs (Zeisel et al., 2018), that have been detected in dopaminergic progenitor grafts (Tiklova et al., 2020) as well as in midbrain organoids (Fiorenzano et al., 2021b). While these midbrain-derived VLMCs also express collagen and *PDGFRa*, they lack mesodermal markers and instead express neuronal markers such as *SOX6*, *FABP7*, *SOX2* (Tiklova et al., 2020; Fiorenzano et al., 2021b) (Supplementary Figure S3J,K).

Additionally, we projected our scRNAseq dataset onto a four-dimensional (4D) reference atlas of a mouse brain (Fleck et al., 2021) in order to spatially and temporally annotate the emerging cell types within silk and non-silk cerebral organoid cultures during development. Interestingly, we found a high correlation of both organoid datasets with the transcriptome of E13.5 mouse forebrain, suggesting a regional identity of our 3D cultures along the rostro-caudal axis. (Supplementary Figure S3L).

We next determined the relative proportion of the identified cell types in silk and non-silk organoids. Consistent with immunohistochemical and functional assessments, scRNAseq revealed a greater percentage of neuronal cells in silk organoids than in non-silk cultures after 4 months, accompanied by an enrichment of the Gene Ontology (GO) term *Neuron differentiation* in silk organoids (Figures 4C,E). This difference in cell-type ratio was also evident when we analyzed the overall expression level of neuronal (*MAP2, SYT1, NCAM1*) and non-neuronal (*COL1A1, DCN, FBLN1*) markers in both 3D culture condition (Supplementary Figure S3M). Interestingly, the cells with fibroblast-like signature and mesoderm origin were almost completely absent in the silk-cerebral organoids (Figures 4A–C).

Importantly, high-resolution analysis investigating only the neuronal compartment (light and dark blue clusters) in both 3D culture systems revealed a higher enrichment score in silk organoids for the following GO terms: *neuron maturation*, *neuron-neuron synaptic transmission*, *neuron migration*, *post-synapse assembly*, and *neurotransmitter secretion* (Figure 4F). In line with electrophysiology data, the increased expression of mature and post-mitotic neuronal markers (*SYT1, NNAT, VAMP2*) as well as voltage-gated potassium, sodium, and calcium channel subfamily members (*KCNB1, SCN3A, CACNG4*) (Figure 4G and Supplementary Figure S3N) also showed that a greater degree of neuronal maturation had been reached within silk-engineered cerebral organoids.

### Silk scaffolding attenuates oxidative stress and interior hypoxia in human brain organoids

**FIGURE 5:**
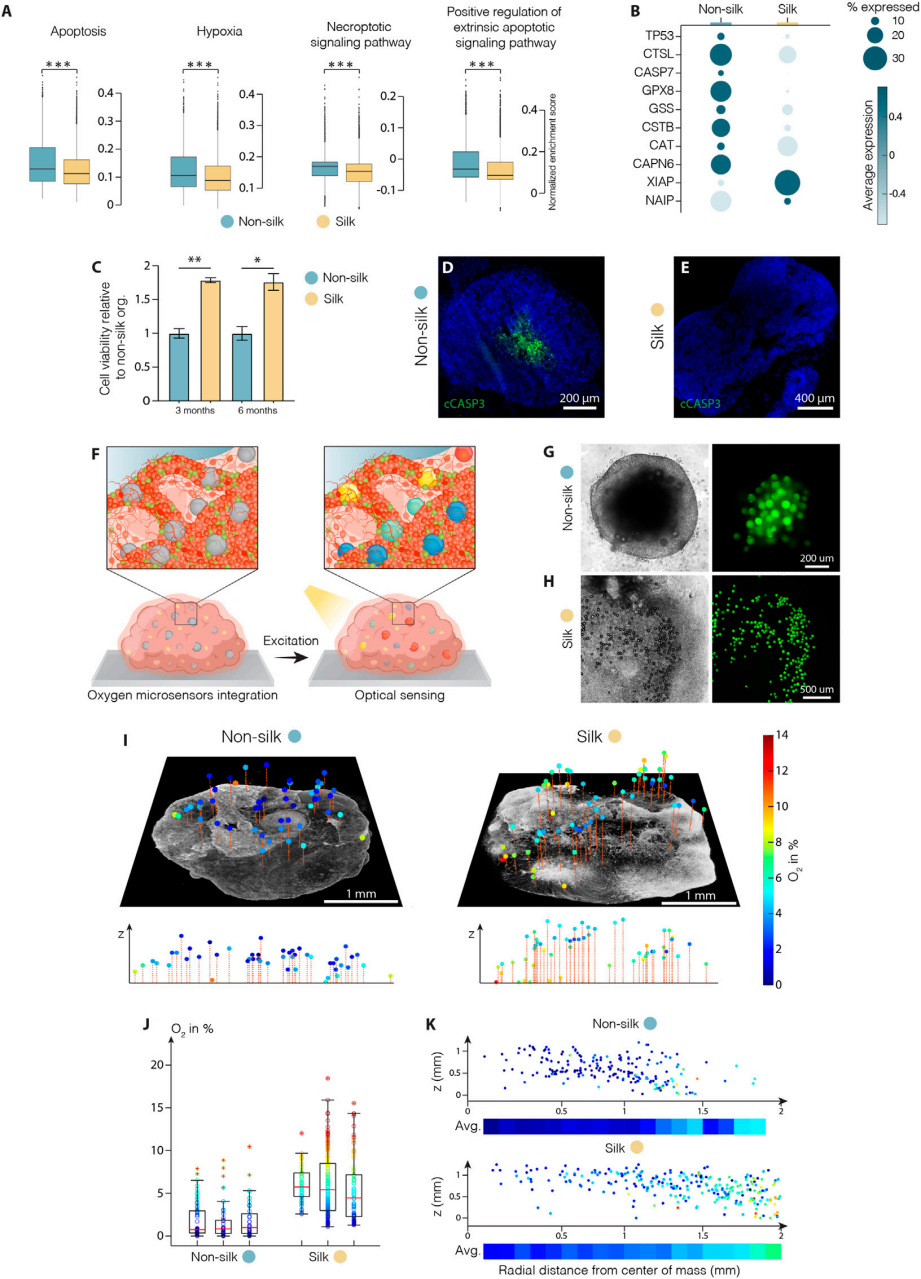
Silk scaffolding increases intra-organoid cell survival. **(A)** Apoptosis, hypoxia, necrosis signalling pathway analysis and **(B)** representative markers of cell death regulation in cerebral organoids grown with and without silk scaffold at 4 months ***p < 0.001, two-tailed Wilcoxon rank sum test. **(C)** Cell viability in cerebral organoids grown with and without scaffold measured by MTT assay and expressed as relative fluorescence units (RFU). Data represent mean ± SEM, normalized over non-silk organoids (n = 3). *p < 0.05, **p < 0.01, two-tailed Mann Whitney test. **(D,E)** Immunohistochemistry of cleaved caspase 3 in non-silk **(D)** and silk **(E)** organoids at day 120. Scale bars 200 µm **(D)** and 400 μm E). **(F)** Schematic illustration of oxygen tension measurements in human brain organoid. **(G,H)** Representative bright-field images of non-silk **(G)** and silk **(H)** brain organoids with integrated CPOx-50-Ptp oxygen sensor beads 8 days after seeding. Scale bars 200 μm G) and 500 μm H). **(I)** 3D oxygen maps of non-silk and silk organoids with simultaneously acquired co-localized live cell images and their cross-sectional oxygen distribution. Scale bars 1 mm. **(J)** Oxygen distribution in 3 silk and non-silk organoids at day 60 showing average oxygenation (red line) and 25th and 75th percentile (box). **(K)** Ratio of oxygen tension to height and distance from organoid center axis in silk and non-silk organoids, and heatmap of average oxygen level for every 100 µm step (n = 3). Nuclei were stained with DAPI.

The reduced intra-organoid variability accompanied by a higher proportion of functionally mature neurons observed in silk-engineered culture suggested that more favorable growth culture conditions were recreated enabling 3D organoid differentiation and mature cell-type specification. We exploited the scRNAseq dataset to analyze the metabolic profiles and compare cell stress response pathways in silk and non-silk organoids. We found ectopic activation of cell stress response signaling only in non-silk generated organoids, highlighting a substantial alteration of metabolic activities (Figure 5A and Supplementary Figure S4A). Specifically, in non-silk organoids, we observed a larger number of cells enriched in hypoxia- and oxidative stress-associated genes, as well as the deregulated expression of enzymatic and molecular cues relating to apoptosis, DNA damage (*TP53*, *CASP7*) and repair (*XIAP, NAIP*) (Figure 5B). These findings suggested insufficient oxygen and nutrient supply within non-silk organoids, leading to several deleterious effects including necrosis. Additionally, ectopic expression of regulatory genes in DNA damage and repair pathways were also found in non-silk organoids (Figures 5A,B and Supplementary Figure S4A). Cell viability analyses (Figure 5C and Supplementary Figure S4B,C) and cleaved-Cas3 immunohistochemistry analysis at month four confirmed the presence of an evident necrotic inner core in conventionally generated organoids, not present in silk bioengineered cultures. (Figures 5D,E).

These findings led us to speculate whether reduced interior cell death was due to the fact that oxygen were uniformly delivered throughout the 3D silk scaffolds. Further, the presence of interconnected micropores structures may improve oxygen diffusion within organoids, thus increasing cell survival and sustaining differentiation. To test this hypothesis, we used phosphorescent microbeads as optical sensors to obtain 3D maps of oxygen tension distribution in silk and non-silk organoids (Figure 5F). The method allows non-invasive microscopy-based sensing of oxygen levels in 3D space by measuring oxygen-mediated quenching of phosphorescence (Wesseler et al., 2022). Oxygen sensitive microbeads were incorporated within human brain organoids at early stages of cell culture in order to achieve homogeneous distribution of microsensors throughout the body of the organoid and prevent their aggregation in the superficial layers during organoid expansions (Figures 5G,H). After 2 months in culture, oxygen tension levels were successfully extracted from beads distributed throughout the volume of both 3D culture systems (Figure 5I). Presence of gradual increase in oxygen levels (∼5%) in the outward radial direction confirmed the competing effects of inward diffusion and cellular consumption within brain organoids. (Figures 5I,K and Supplementary Figure S4D). The overall mean oxygen level was significantly higher in silk than in non-silk organoids: non-silk organoids exhibited oxygen tensions of approximately 1.9 ± 0.7% dissolved O_2_ (DO) compared to 5.8 ± 1.6% DO in silk brain organoids (mean ± S.E.M., Figure 5J). Importantly, comparison of spatial distribution of oxygen tension also showed that the silk scaffolding strategy resulted in higher, more homogeneous, and physiological tissue oxygenation in all areas of the organoid (Figure 5K and Supplementary Figure S4D). These results indicate that microarchitecture facilitated by silk scaffolding significantly improves organoid oxygenation, therefore increasing cell survival and leading to uniform cellular and functional intra-organoid composition.

## Discussion

Pioneering studies in the last decade (Eiraku et al., 2011; Lancaster et al., 2013) revealed the remarkable ability of hPSCs to self-pattern and self-organize *in vitro,* generating complex neural tissue structures that contain a broad range of cell types mimicking several brain regions. Since then, unguided brain organoid differentiation protocols have been used extensively to model neurodevelopmental processes and neurodegenerative diseases in more physiologically relevant conditions than previously possible (Lancaster et al., 2013; Quadrato et al., 2017; Madhavan et al., 2018; Pellegrini et al., 2020). However, the organoid field is still riddled with issues of reproducibility and variability in terms of internal organization and cellular composition (Bhaduri et al., 2020; Qian et al., 2020; Fiorenzano et al., 2021c). Despite the obvious necessity to alleviate these issues, adequate multidisciplinary routes remain largely unexplored. To address this need, we developed a bioengineering approach based on silk scaffolds for the generation of cerebral organoids with reduced intra-organoid variability and enhanced functional maturation. We demonstrated that a recombinant spider silk microfiber network supports unguided hPSC differentiation and spontaneous self-patterning and morphogenesis into more oxygenated and healthier brain organoids.

In contrast to previously presented bioengineering methodologies for the establishment of brain organoid scaffolding, the formation of a silk fiber network does not require the laborious manual isolation of individual fibers (Lancaster et al., 2017) or specialized equipment such as a 3D printer (Rothenbucher et al., 2021). Our simple scaffold generation approach is easily executable by other research groups and is compatible with potential high-throughput applications. Spider silk fiber technology present several advantages over other scaffold-based methods. Spider silk fibers 1) form a strong and elastic 3D structure to guide the arrangement of hPSCs into an organ-like configuration; 2) can be functionalized with specific bioactive molecules to help drive the differentiation of hPSCs into specific human tissues; 3) act as an anchored scaffold at the initial stage of differentiation, thus facilitating hPSC seeding and adhesion to the microfibers; and 4) create a microporous structure promoting the intraorganoid delivery of growth factors.

Furthermore, recombinant spider silk protein provides a natural, highly biocompatible, and molecularly defined material source. Importantly, the silk scaffold polymerized around air bubbles forms a stable structure with an intricate hierarchical architecture. On the macroscale, the scaffold takes the shape of a robust framework that allows reproducible cell seeding and expansion in the 3D space; on the microscale, silk polymerizes into flat microfibers with a nanoscale grass-like texture that provides cell-interactive topological cues further enhanced by the presence of human recombinant laminin. Easily obtainable and structurally defined across multiple length scales, the engineered silk scaffold presents a unique biomaterial support for cerebral organoid formation.

One of the main issues with the conventional brain organoid methodologies is the lack of a vascular network, resulting in incomplete and heterogeneous differentiation caused by the restricted diffusion of oxygen and nutrients as well as the accumulation of metabolic waste in interior layers (Pasca et al., 2019). Silk scaffolding mitigates detrimental hypoxic effects by facilitating the formation of an interconnected microcavity network within the organoid that not only increases surface-to-volume ratio (by exposing a larger fraction of cells to nutrients and oxygen) but also creates an interpenetrating system for metabolic waste removal. As such, the silk bioengineering approach reduces spontaneous differentiation toward non-neuroectodermal populations and prevents the emergence of resilient stem cell niches at early stages of organoid formation. In comparison to the conventional methodologies, our silk scaffolding strategy leads to more homogeneous differentiation both temporally (cells synchronously exit pluripotency and enhance neuroectoderm formation) and spatially (absence of localized populations with undesirable cell types), underscoring the supportive role of the scaffold during hPSC self-patterning (Xu et al., 2015; Fiorenzano et al., 2016). At early stages of silk organoid differentiation, neural stem cells are enclosed in rosette niches, as in conventionally generated organoids. However, a difference in PAX6^+^ cell organization surrounding the central lumen is detected, possibly reflecting a greater level of maturation in silk organoids. At later stages, silk organoids exhibit the layering organization of cortical tissue, including the formation of a polarized cortical plate. Neurons show rudimental spatial separation into an early-born inner layer (CTIP2^+^) and a late-born outer layer (SATB2^+^). However, this layering organization remains limited, suggesting that additional cues and growth factors are needed to generate the complex stereotypical organization of layers II–VI *in vitro*. By combining silk scaffolding with the most recent and sophisticated guided differentiation protocols, it may be possible to recapitulate a similar degree of spatial organization of the mature layer as *in vivo*.

scRNAseq revealed a larger fraction of neuronal cells in silk than in non-silk organoids at the expense of populations of meso-endodermal origin, in particular those with endothelial identities. In line with functional assessments, neurons in silk organoids exhibited more mature molecular features with a higher expression of post-mitotic synaptic markers and ion channel subfamily members at single cell resolution. Importantly, the silk-bioengineered approach does not alter organoid differentiation trajectories but instead gives rise to a more robust and reproducible neuroectodermal signature than that of non-silk organoids, with the absence of pluripotent and meso-endodermal cell type contaminants.

This work presents multiple technological advantages associated with the use of silk scaffolding. Firstly, because the silk scaffolds are initially anchored at the bottom of the plate, cell distribution along the fibers can be finely controlled to overcome cell seeding discrepancies associated with free-floating scaffolds. The amenable nature of the silk technology could also allow the generation of brain organoids with specific size and shape by introducing molding and varying cellular density as well as silk fiber volume fraction. The optical oxygen sensing technique presented here provides a powerful approach for both temporal and spatial oxygen mapping in brain organoids that could be applied to other organoid protocols with the aim of determining and enhancing tissue oxygenation in these model systems or tuning them to mimic native tissue oxygen levels (Kajtez et al., 2021).

It is important to note that our approach is compatible with bioengineering solutions that seek to advance other aspects of organoid formation. For example, silk-based organoids would be compatible with microfluidic systems or engineered bioreactors. Furthermore, since silk scaffolding forms a secondary polymer network (in addition to the embedding hydrogel network), our platform is adaptable to other bioengineering strategies based on introducing matrigel substitutes with specific components that mimic the native brain extracellular matrix more closely. These efforts have yielded several studies where brain organoids were embedded in collagen, hyaluronic acid, or decellularized brain extracellular matrix, which could all be combined with silk (Lindborg et al., 2016; Zafeiriou et al., 2020; Cho et al., 2021; Simsa et al., 2021). Merging multiple bioengineering solutions together could lead to hybrid approaches that provide much needed advancements in the field of brain organoids. In this regard, silk scaffolding provides a versatile approach that can also be applied to guided differentiation of organoids into other brain regions, such as ventral midbrain (Fiorenzano et al., 2021b).

In summary, the silk bioengineering methodology presented here gives rise to cerebral organoids characterized by homogeneous 3D differentiation, a reduction in both metabolic stress and the formation of a necrotic core, more complex cytoarchitectural features, and improved functional maturation. Our approach therefore provides a novel platform based on spontaneous hPSC differentiation that can be used to better study key aspects of human brain physiology in a dish and, ultimately, in drug discovery and regenerative medicine.

## Materials and methods

### Human pluripotent stem cell culture

Undifferentiated human pluripotent stem cells (hPSCs) RC17 (Roslin Cells, hPSCreg RCe021-A) were cultured on 0.5 μg/cm^2^ Lam-521 (BioLamina, LN-521)-coated plates in iPS-Brew XF medium (Miltenyi, 130-104-368). Once they reach confluence, the cells were passaged with 0.5 mM EDTA (for 7 min at 37°C, Thermo fisher Scientific, 15575020) and seeded at a density of 2,500-10,000 cells/cm^2^ in iPS Brew medium with the addition of 10 μM Y-27632 (Miltenyi, 130-106-538) for the first 24 h after passaging.

### Human cerebral organoid differentiation

To start 3D organoid formation, homogeneous hPSC culture at 75-90% confluency of the well area was washed with DPBS (- ${Mg}^{2+}$ /- ${Ca}^{2+}$ , Thermo Fisher Scientific, 14200075) and incubated with Accutase solution (50 μL/cm^2^, Thermo Fisher Scientific, A1110501) for 5 min at 37°C. The cells were then centrifuged, resuspended in mTeSR1 (Stem Cell Technologies, 05850) with 10 μM Y-27632 and counted. To generate non-silk organoids, single cell suspension was then seeded at a density of 8,000 cells/well in an ultra-low attachment 96-well plate (Costar, round bottom, REF 7007) and supplemented with mTeSR1 with 10 μM Y-27632 to reach a final volume of 25 μl/well as reported in Lanc et al. (Lancaster and Knoblich, 2014). After 2 days of culture, 50% of media was replaced with mTesR1 without the Y-27632 supplement. After three additional days, differentiation medium consisting of DMEM/F12 (Thermo Fisher Scientific, 21331020), 1:100 N2 supplement (Thermo Fisher Scientific, 17502048) and 2 μg/ml Heparin (Sigma-Aldrich, H3149) was added from day 0-6 and replaced every second day. 1% minimum essential medium non-essential amino acids (MEM-NEAA; Sigma-Aldrich, M7145), 0.1% 2-mercaptoethanol (Merck, 8057400005), 1:500 penicillin-streptomycin (Thermo Fisher Scientific, 15140122), and 200 mM L-glutamine (Thermo Fisher Scientific, 25030081) were maintained for the entire differentiation period, as reported in Lancaster et al. (Lancaster and Knoblich, 2014). For the first 48 h the medium was also supplemented with 3% Knockout Serum Replacement (KSR, Thermo Fisher Scientific, 10828010). At day 8 each organoid was transferred in a sterile dimple, embedded evenly in 30 uL of matrigel (Corning, 354234) and incubated at 37°C for 25 min. After the polymerisation of matrigel was complete, the organoids were transferred in an ultra-low attachment six well plate (Corning, flat bottom, REF 3471) with 4 ml/well of 1:1 DMEM/F12:Neurobasal medium (Thermo Fisher Scientific, 21331020 and A3582901), 1:200 N2 supplement, 1:100 B27 supplement (Thermo Fisher Scientific, 12587010) and 2.5 μg/ml insulin (Sigma-Aldrich, I9278-5 ML), as reported in Lancaster et al. (Lancaster and Knoblich, 2014). The medium was replaced every second day. At day 15, the medium was substituted with 1:1 DMEM/F12:Neurobasal, 1:200 N2 supplement, 1:50 B27 + Vitamin A supplement (Thermo Fisher Scientific, 17504044) and 2.5 μg/ml insulin solution supplemented with 400 μM Ascorbic Acid (Sigma-Aldrich, A4403-100 MG). For terminal differentiation after day 25, the previously described medium was supplemented with 400 μM Ascorbic Acid, 10 ng/ml BDNF (Miltenyi, 130-096-286), 10 ng/ml GDNF (R&D Systems, 212-GD-010) and 200 μM cAMP (Sigma-Aldrich, D0627-1G).

### Generation of bioengineered silk cerebral organoids

A 20 μL droplet of prepared Biosilk solution (BioLamina, BS-0101) functionalized with 5 μg/ml Lam-111 (BioLamina, LN111) and 10 μM Y-27632 was placed in the centre of hydrophobic cell culture wells of a 24-well plate (Sarstedt, REF 833922500) (Fiorenzano et al., 2021b). Air bubbles were introduced into the droplets by pipetting up and down (10-15 strokes) creating a dense foam. Human pluripotent stem cells were detached using Accutase solution (50 μl/cm^2^) and prepared as a single cell suspension at high concentration (15,000 cells/μl in mTeSR1 with 10 μM Y-27632). A total of 75,000 cells was then added to the silk foam, dispersed homogeneously, and stabilized at 37°C for 25 min. Silk bioengineered culture was then incubated with 2 ml of mTeSR1 with 10 μM Y-27632 for 5 days and then cultured following the cerebral differentiation protocol as described in Lancaster et al. (Lancaster and Knoblich, 2014). 8 days after the start of differentiation, silk organoids were lift up with a scalper and detached from the bottom. Silk cultures were then embedded in matrigel as described above and grown in suspension (free floating) in a 6-well plate (Corning, 3,471) thereafter.

### Silk and live-cell staining and microscopy

To prepare it for fluorescence imaging, silk scaffold was polymerized in a well with glass bottom compatible with high resolution imaging. The scaffold was then incubated for 30 min in 0.5% w/w Rhodamine B solution (Sigma Aldrich, 83689) in PBS and rinsed thoroughly to remove the excess dye. On separate samples, in order to visualize live cell distribution on the silk scaffold 10 days after cell seeding, cell-laden scaffolds were incubated with 2 µM Calcein AM (ThermoFisher Scientific, C3100MP) for 30 min at 37°C and then rinsed thoroughly with cell culture medium. Imaging was performed on a Nikon A1RHD confocal microscope equipped with a 20x objective. Image processing was performed in NIS-elements software (Nikon).

### Scanning electron microscopy

Samples were fixed in 2% buffered glutaraldehyde (Sigma-Aldrich, G6257-10 ML) overnight and then rinsed in 0.1% HEPES buffer. Postfixation was performed for 1 h in 2% osmium tetroxide (TedPellaInc, n°18450, 18451, 18456). Samples were dehydrated in ethanol dilution series, then exposed to dilution series of hexamethyldisilazane (HMDS; TedPellaInc, n°18605) and left to dry in a fume hood overnight. Samples were mounted on 25 mm aluminum stubs, sputter coated with 10 nm Platinum/Palladium (80:20) and imaged in a Jeol JSM-7800F scanning electron microscope.

### Organoid cryosectioning and immunofluorescence

Organoids were fixed with 4% PFA for 5 h at room temperature and then left to sink in sucrose 30% solution + OCT cryomount (volume ratio 1:1) overnight at 4°C. Organoids were then transferred to a cryomold filled with OCT, frozen in dry ice and stored at −80°C. After at least 24 h at −80°C, mounted organoids were cut in 20 μm slices using a cryostat and the slides were stored at −20°C until needed.

For the immunofluorescence, sections were dried for 5 min at room temperature, then washed with KPBS 1X three times and fixed again with PFA for 10 min at room temperature. After washing other three times, the slides were then incubated in blocking solution containing KPBS 1X +0.3% Triton solution+ 5% serum of secondary antibody host species for at least 1 h at room temperature. Primary antibodies (see Supplementary Table S1) in blocking solution were added to the slides for an overnight incubation at 4°C. Sections were then washed, incubated with appropriate secondary fluorophore-conjugated antibodies (Alexa Fluor 488, 594, 647 used at 1:400, Jackson ImmunoResearch Laboratories) + DAPI (1:1,000) in blocking solution for 1 h at room temperature and mounted on glass coverslips using FluorSave Reagent.

### Microscopy

Brightfield images were taken using an Olympus CKX53 inverted microscope with a 4x objective (UPlanFL N 4x/0.13 NA) and acquired with the OLYMPUS cell Sens Standard v2.3 software. Fluorescent images were captured within a week from staining using a Leica DMI6000B widefield microscope or a Leica TCS SP8 laser scanning confocal microscope. The image acquisition software was Leica LAS X and images were processed using Adobe Photoshop CC 2020. In comparison experiments, all the images were taken with the same software settings and any adjustments were applied equally across the entire image, without the loss of any information.

### qRT-PCR

Total RNAs were isolated on day of analysis from three individual organoids/sample using the RNeasy Micro Kit (Qiagen, 74004), according to manufacturer instructions. Reverse transcription was performed using Maxima First Strand cDNA Synthesis kit for qRT-PCR (Thermo Fisher, K1641). cDNA was then mixed with the relevant primers (0.95 μM; Integrated DNA Technologies, see Supplementary Table S2) and SYBR Green Master mix (Roche) using a Bravo pipetting robot instrument (Agilent). The analysis was performed with a LightCycler 480 II instrument (Roche) using a 40x cycle two-step protocol with a 60°C, 1 min annealing/elongation step and a 95°C, 30 s denaturation step. The average CT values were calculated from three technical replicates and were used to determine the relative gene expression using the ΔΔCT method. The average fold change was normalized against two different housekeeping genes (ACTB and GAPDH) and the results were given as relative gene expression over undifferentiated hPSCs.

### Cell viability analysis

Cell viability was measured using the MTT cell proliferation assay kit (Abcam, ab211091), following manufacturer instructions on organoids dissociated with papain after 3 and 6 months of culture. Absorbance was analyzed at 620 nm, using 100,000 cells/sample harvested from three organoids per condition. Each organoid was analyzed in three to six replicates in a 96 well plates using Biochrom Asys Expert 96 Microplate reader (Biochrom). DNA damage was detected *via* Click-iT plus Tunel assay (Invitrogen C10617) according to the manufacturer´s instructions and a positive control incubated with 1 unit of DNAse I for 30 min at RT, was included.

### Bioinformatics analysis of bulk RNA sequencing data

cDNA libraries were prepared using the Illumina TruSeq library preparation kit and sequenced with 2 × 150 bp paired end reads on an Illumina NextSeq 500 High Output kit. Raw base calls were demultiplexed and converted into sample specific fastq format files using default parameters of the bcl2fastq program provided by Illumina (v. 2.19). Resulting fastq files were quantified using Salmon (1.1) using Gencode version 33 as the gene model. Downstream analysis was performed in R using DESeq2 (1.3) for differential expression analysis and EnhancedVolcano (1.8) for plotting volcano plots. Gene ontology analysis was performed using the gProfiler web tool (database updated on 07/05/2021).

### Library preparation, and raw data processing for single cell RNA sequencing

For 10x Genomics single-cell RNA sequencing (scRNAseq), organoids at day 120 were prepared as a single cell suspension with the Papain kit (Worthington, #LK003150). Single cells were loaded onto 10x Genomics Single Cell 3′Chips along with the master mix following the manufacturer´s instruction for the Chromium Single Cell 3′ Library to generate single cell gel beads in emulsion (GEMs, version three chemistry). The resulting libraries were sequenced on a NovaSeq in a 2 × 100 bp paired end mode. Pre-processing of sequencing data was conducted with the Cell Ranger pipeline (10x Genomics, Cell Ranger version 6.0) with alignment to GrCh38 (2020-A) in order to generate the matrix files used for bioinformatic analysis.

### Bioinformatics analysis of single cell RNA sequencing data

Downstream analysis of scRNAseq matrix files was conducted with Seurat (v 4.0.6 and R version 4.1.0). Single cell expression values were normalized, and the top 2000 highly variable genes were identified using *vst*. Data were then scaled, and principal component analysis (PCA) was performed using 30 dimensions. Cells with less than 200 or more than 5,000 genes detected were filtered out from further analyses, as well as cells with more than 10% mitochondrial reads. Data integration between different samples was performed with Harmony algorithm, which converged after seven iterations. The top 25 principal components were used for clustering using Seurat´s FindNeighbors and FindClusters functions (Louvain algorithm, resolution 0.1). Clusters identities were assigned based on cluster gene markers as determined by Seurat´s FindAllMarkers function (minimum percentage of cells expressed = 0.25 and log fold change threshold = 0.25) together with gene expression values of known markers. Differential gene expression analysis between samples was carried out using the Wilcoxon rank sum test and genes with an FDR-corrected *p*-value < 0.05 were considered significant. Density plots were generated using the plot_density function from the “Nebulosa” package (v 1.2.0). Comparison with published dataset was performed by projecting reference labels onto scRNAseq data using Seurat’s v4 label transfer functions. Spatial similarity maps of silk and non-silk brain organoids with E13.5 mouse embryos (Allen Brain Atlas) were generated using VoxHunt package (v1.0.1; 300 genes). Gene Set Enrichement Analysis was performed using the “escape” package (v1.2) and gene sets obtained from MSigDB collections.

### Electrophysiology

Whole cell patch-clamp electrophysiological recordings were performed on matrigel embedded organoids maintained in a Krebs solution gassed 95% O_2_—5% CO_2_ at room temperature that was changed every 20 min. The composition of the standard solution was (in mM): 119 NaCl, 2.5 KCl, 1.3 MgSO₄, 2.5 CaCl₂, 25 Glucose and 26 NaHCO₃. For recordings Multiclamp 700B amplifier (Molecular Devices) was used together with borosilicate glass pipettes (3-7 MOhm) filled with the following intracellular solution (in mM): 122.5 potassium gluconate, 12.5 KCl, 0.2 EGTA, 10 Hepes, two MgATP, 0.3 Na₃GTP and eight NaCl adjusted to pH 7.3 with KOH as reported in Fiorenzano et al. (Fiorenzano et al., 2021b). Data acquisition was performed with pClamp 10.2 (Molecular Devices, San Jose, CA, United States); current was filtered at 0.1 kHz and digitized at 2 kHz. Cells with neuronal morphology and round cell body were selected for recordings. Resting membrane potential was monitored immediately after breaking-in in current-clamp mode. Thereafter, cells were kept at a membrane potential of −65 to −70 mV, and 500 ms currents were injected from −20 pA to +35 pA with 5 pA increments to induce action potentials. For inward sodium and delayed rectifying potassium current measurements cells were clamped at −70 mV and voltage-depolarizing steps were delivered for 100 ms at 10 mV increments. Spontaneous activity was recorded in voltage clamp mode at −70 mV. Data were analysed using the software Clampfit 10.3 (Molecular Devices, San Jose, CA, United States) and Igor Pro 8.04 (Wavemetrics, Portland, OA, United States) with the NeuroMatic package (Rothman and Silver, 2018).

### Oxygen sensing

To obtain 3D maps of oxygen tension distributions in cerebral organoids during culture, a recently presented 3D optical oxygen mapping method based on tissue integrated oxygen microsensor beads mapped by phosphorescence lifetime microscopy, described in detail in our previous publication (Wesseler et al., 2022), was adapted for organoid cultures. To generate conventional brain organoids, oxygen sensor microbeads (0.5 mg/ml CPOx-50-Ptp, Colibri Photonics GmbH, Germany) were mixed with single cell suspension of hPSCs (160k cells/mL), seeded in an ultra-low attachment 96-well plate (50 µl suspension/well, Costar, round bottom, REF 7007) and supplemented with mTeSR1 with 10 μM Y-27632 to reach a final volume of 250 μl/well. To generate silk organoids, a single cell suspension at higher concentration was prepared (15,000 cells/μL in mTeSR1 with 10 μM Y-27632, 2.5 mg/ml CPOx-50-Ptp) and dispersed homogeneously throughout the silk scaffold. Both organoid systems were then differentiated as described in the section “Human cerebral organoid differentiation”. For co-localized imaging of live cells and oxygen sensor microbeads, organoids were stained with 2 μg/ml calcein AM in terminal differentiation medium prior to optical oxygen mapping. Imaging analysis and visualization was performed with custom programmed Matlab software (Mathwork, United States), available upon request through aforementioned publication.

### Statistics and reproducibility

Statistical analysis of qRT-PCR data was performed using two-tailed unpaired *t*-test and *p*-values<0.05 were considered significant. Data were statistically analyzed with the GraphPad Prism nine software and presented as mean ± SEM except where stated otherwise. For box-plots representing oxygen distribution in individual organoids, the average value, 25th - 75th percentiles and minimum – maximum values are represented as red line, box and whiskers respectively. Statistical analysis of sequencing data was conducted using two-tailed Wilcoxon rank-sum test (Seurat v4) in R v4.1.0. For all figures: *p < 0.05, **p < 0.01, ***p < 0.001. Immunohistochemical staining images are representative of 6–12 sections from at least four biologically independent organoids.

## Data Availability

The datasets presented in this study can be found in online repositories. The names of the repository/repositories and accession number(s) can be found below: https://www.ncbi.nlm.nih.gov/geo/, GSE196423, https://www.ncbi.nlm.nih.gov/geo/, GSE202993.
