# Supplementary figures and images for: Silk scaffolding drives self-assembly of functional and mature human brain organoids
Source: Front Cell Dev Biol. 2022 Oct 14;10:1023279. doi: 10.3389/fcell.2022.1023279 (PMC9614032; doi:10.3389/fcell.2022.1023279)

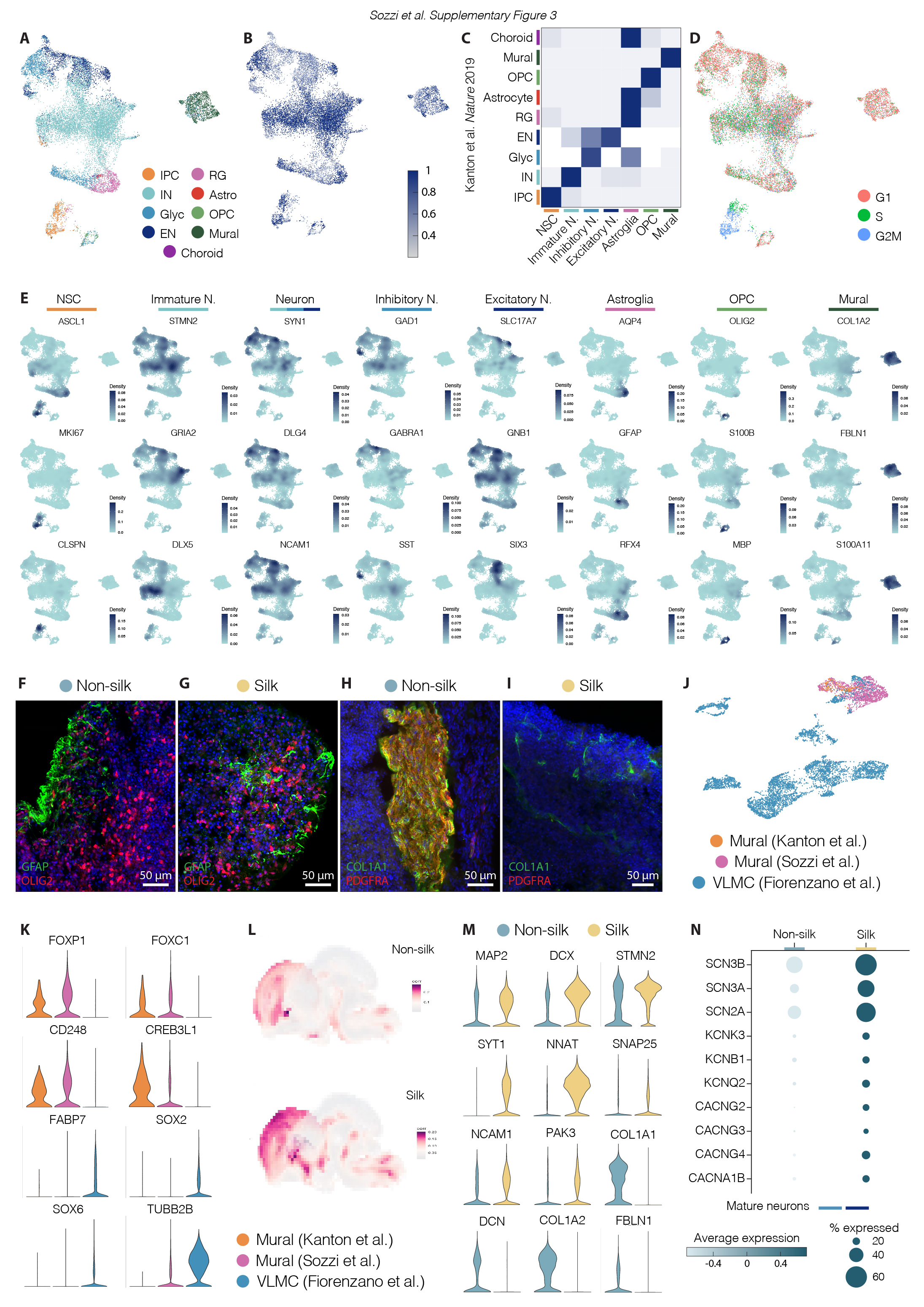

Supplement: Supplementary file 1 [file Image3.JPEG]

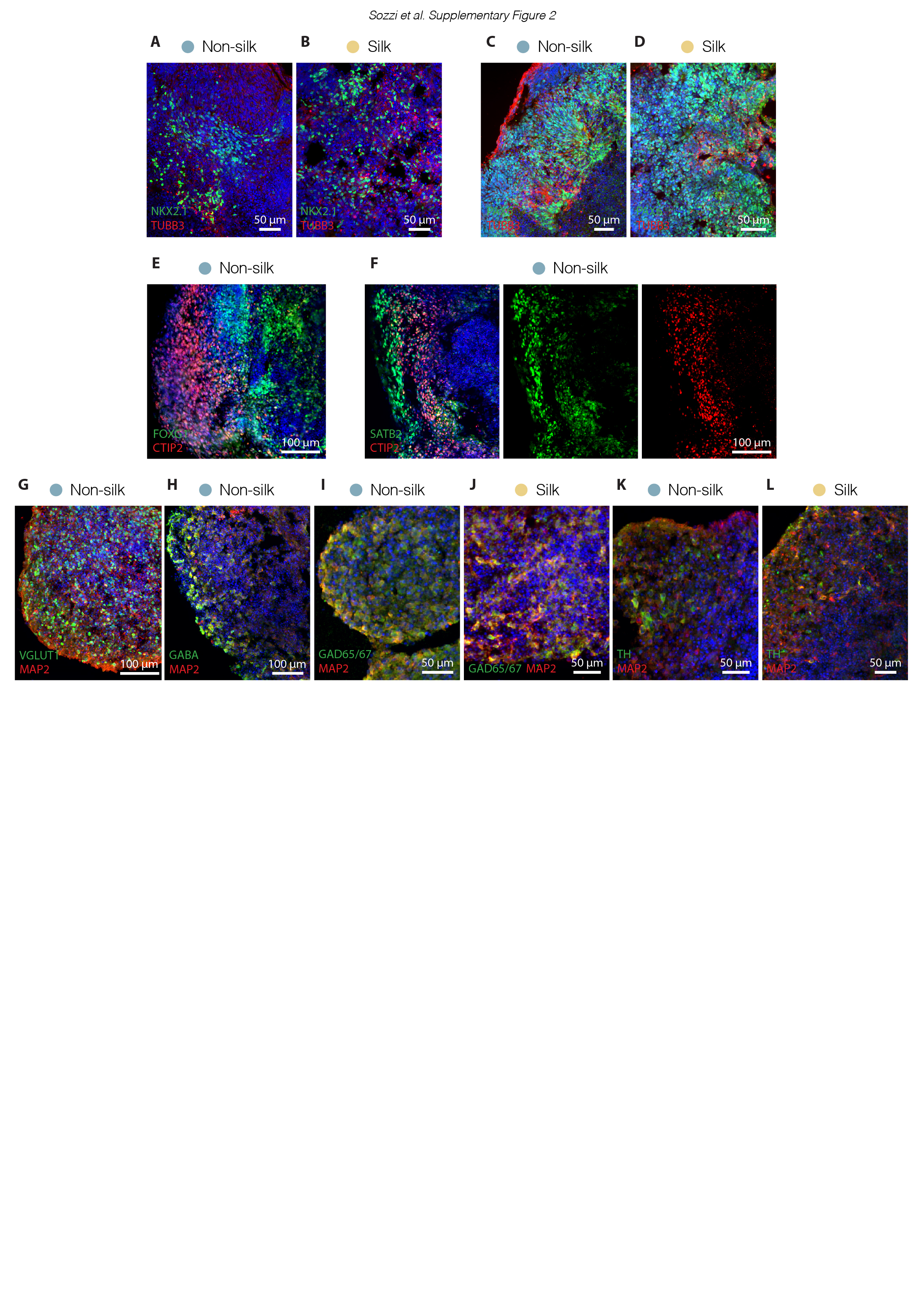

Supplement: Supplementary file 2 [file Image2.jpg]

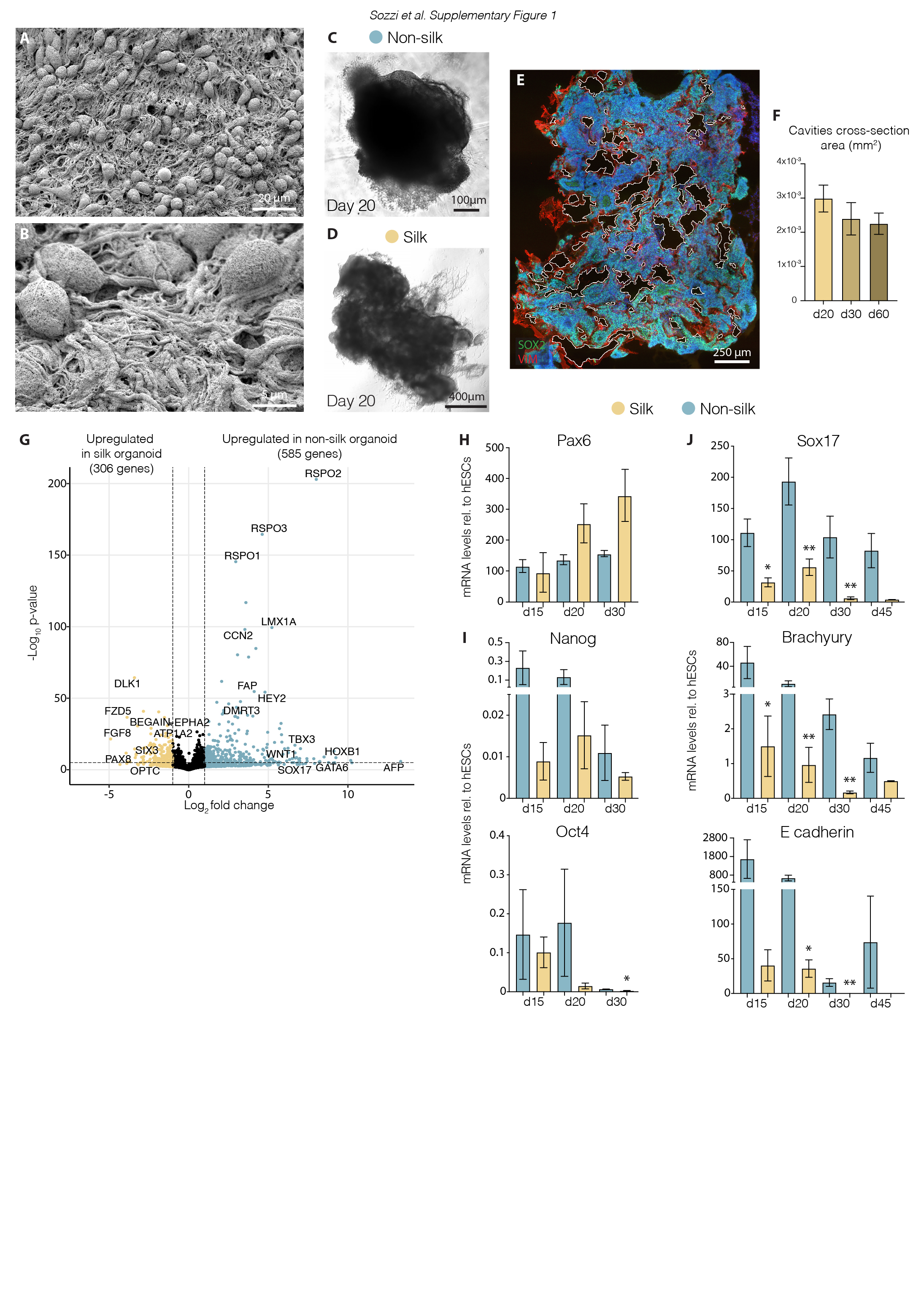

Supplement: Supplementary file 3 [file Image1.JPEG]

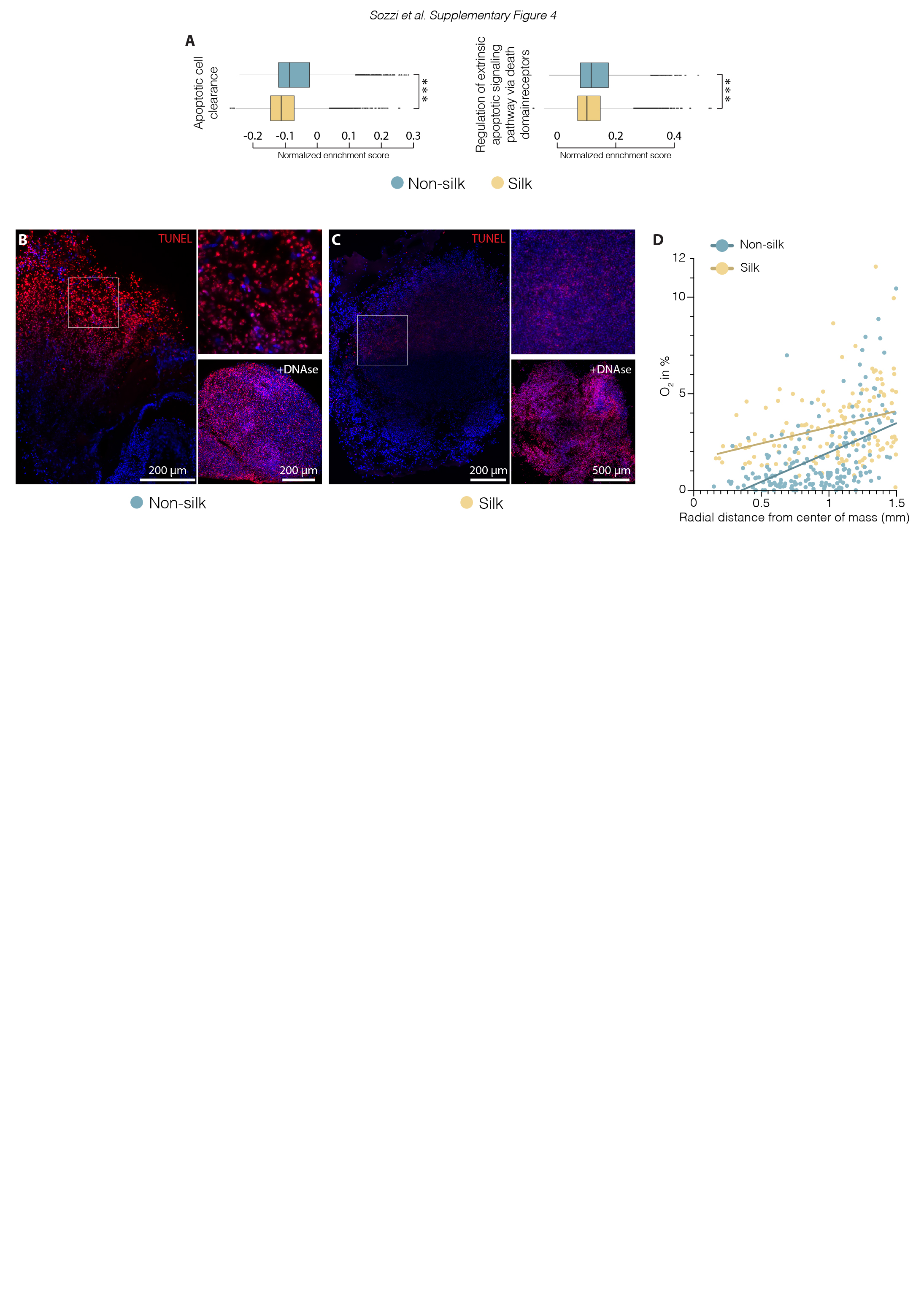

Supplement: Supplementary file 4 [file Image4.JPEG]
