# Supplementary material for: Silk scaffolding drives self-assembly of functional and mature human brain organoids
Source: Front Cell Dev Biol. 2022 Oct 14;10:1023279. doi: 10.3389/fcell.2022.1023279 (PMC9614032; doi:10.3389/fcell.2022.1023279)
Supplement: Supplementary file 5 [file DataSheet1.docx]

Supplementary Material

## Supplementary figures legends

**Supplementary Figure 1.** **A,B**) Scanning electron images (SEM) images showing neuronal cell morphology and network formation within cerebral organoids at day 60. Scale bars 20 μm A) and 5 μm B). **C,D**) Representative bright-field images of non-silk C) and silk D) brain organoids at day 20. Scale bars 100 μm C) and 250 μm D). **E**) Immunohistochemistry of SOX2/VIM in silk organoid at day 20 with internal cavities highlighted in white. Scale bar 250 μm. Nuclei were stained with DAPI. **F**) Cross sectional area of the cavities in silk brain organoids over time. Data represent mean ± SEM obtained from 3 individual organoids per time-point. **G**) Volcano plot of statistical significance versus fold change of differentially expressed genes (p <0,05, log2FC >1) between silk and non-silk organoids, with manually selected genes named. **H-J**) RT-qPCR analysis of neuroectodermal marker PAX6 H), stem cell markers NANOG and OCT4 I) and non-neural lineage markers SOX17, Brachyury and E-Cadherin J) during cerebral organoid differentiation. Values are given as fold change relative to undifferentiated hPSCs. * p<0.05, ** p<0.01, two-tailed unpaired t-test.

**Supplementary Figure 2. A-D**) Immunohistochemistry of NKX2.1/TUBB3 A,B) and PAX6/ TUBB3 C,D) in organoids grown with or without silk scaffold at day 30. Scale bars 50 μm. **E**) Cryosection of non-silk brain organoid at day 60 showing FOXG1/CTIP2 double staining. Scale bar 100 μm. **F**) Immunohistochemistry of SATB2/CTIP2 showing cortical layer formation in non-silk brain organoid at day 120. Scale bar 100 μm. **G,H**) Cryosections of non-silk brain organoids at day 120 showing VGLUT1/MAP2 G) and GABA/MAP2 H) double staining. Scale bars 100 μm. **I-L**) Immunohistochemistry of GAD65-67/MAP2 I,J) and TH/MAP2 K,L) in organoids grown with or without silk scaffold at day 120. Scale bars 50 μm. Nuclei were stained with DAPI.

**Supplementary Figure 3. A-C**) UMAP plots showing predicted cell types A), prediction score B) and relative overlapping quantification C) using published cerebral organoid dataset as reference (Kanton et al. Nature 2019). IPC, Intermediate progenitor; IN, Immature neuron; Glyc, Glycinergic neuron; EN, Excitatory neuron; RG, radial glia; Astro, Astrocyte; OPC, Oligodendrocyte precursor. **D**) UMAP embeddings showing the predicted cell cycle phase using Seurat CellCycleScoring function. **E**) Density feature plots visualizing specific gene expression across clusters. **F-I**) Immunohistochemistry of F,G) GFAP/OLIG2 and H,I) COL1A1/ PDGFRa in organoids grown with or without silk scaffold at day 120. Scale bars 50 μm. **J**) UMAP plots of mural cells from cerebral organoids (this study, purple, Kanton et al. Nature 2019, orange) and vascular leptomeningeal cells (VLMC) detected in ventral midbrain patterned organoids (Fiorenzano et al. Nature Comm. 2021, light blue). **K**) Expression of representative mesoderm and neurogenesis associated markers in perivascular cell types from distinct datasets. **L**) Spatial similarity maps of silk and non-silk brain organoids generated using VoxHunt with E13.5 mouse embryos (Allen Brain Atlas). Scores were calculated using average gene expressions for all cells using Pearson correlations. **M**) Expression of representative neuronal and vasculature-associated markers in human brain organoid grown with or without silk scaffold after 4 months in culture. **N**) Dot plot showing expression levels of selected genes coding for ion channels in silk and non-silk brain mature neuronal clusters. Nuclei were stained with DAPI.

**Supplementary Figure 4. A**) Gene set enrichment analysis (GSEA) showing a statistically significant different score for apoptotic signalling pathway terms in non-silk and silk brain organoids. *** p<0.001, two-tailed Wilcoxon rank sum test. **B-C**) TUNEL staining of non-silk B) and silk C) organoids after 4 months in culture, including magnification of selected areas (upper panel) and positive control treated with DNAse I for 30min (lower panel). Scale bars 200 μm B), 200 μm C) left and 500 μm C) right. Nuclei were stained with DAPI. **D**) Relation of oxygen tension to distance from organoid center of mass in silk and non-silk organoids, including trendline obtained through simple linear regression.

Table S1.

Primary antibodies

| **Antigen** | **Specie** | **Company** | **Cat. no** | **Dilution** |
| --- | --- | --- | --- | --- |
| BRACHYURY | Goat | R&D Systems | AF2085 | 1:300 |
| Cleaved CASP3 | Rabbit | Cell Signaling | 9661 | 1:500 |
| COL1A1 | Sheep | R&D Systems | AF6220 | 1:500 |
| CTIP2 | Rat | Abcam | AB18465 | 1:500 |
| E-CADHERIN | Mouse | BD Biosciences | 610182 | 1:300 |
| FOXG1 | Rabbit | Novus | NBP1-56594 | 1:300 |
| GABA | Rabbit | Sigma-Aldrich | A2052 | 1:2000 |
| GAD65/67 | Rabbit | Abcam | AB49832 | 1:1000 |
| GFAP | Rabbit | Dako | Z0334 | 1:1000 |
| MAP2 | Chicken | Abcam | AB5392 | 1:2000 |
| NKX2.1 | Rabbit | Abcam | AB133737 | 1:500 |
| OCT4 | Mouse | Santa Cruz | SC5279 | 1:500 |
| OLIG2 | Goat | R&D Systems | AF2418 | 1:500 |
| PAX6 | Rabbit | Abcam | AB195045 | 1:300 |
| PDGFRa | Rabbit | Cell Signaling | 5241S | 1:300 |
| SATB2 | Rabbit | Abcam | AB34735 | 1:300 |
| SOX17 | Goat | R&D Systems | AF1924 | 1:300 |
| SOX2 | Rabbit | Millipore | AB5603 | 1:400 |
| TH | Rabbit | Millipore | AB152 | 1:500 |
| TUBB3 | Mouse | BioLegend | 801202 | 1:1000 |
| VGLUT1 | Rabbit | SYSY | 135303 | 1:500 |
| VIM | Mouse | Dako | M0725 | 1:50 |
| ZO1 | Mouse | Invitrogen | 33-9100 | 1:300 |

Table S2.

Sequence of qPCR primers

| **Gene name** | **Primer Sequence (fwd/rev)** |
| --- | --- |
| ACTB | CCTTGCACATGCCGGAG  GCACAGAGCCTCGCCTT |
| GAPDH | TTGAGGTCAATGAAGGGGTC  GAAGGTGAAGGTCGGAGTCA |
| PAX6 | TGGTATTCTCTCCCCCTCCT TAAGGATGTTGAACGGGCAG |
| NANOG | TTGGGACTGGTGGAAGAATC  GATTTGTGGGCCTGAAGAAA |
| OCT4 | TCTCCAGGTTGCCTCTCACT  GTGGAGGAAGCTGACAACAA |
| SOX17 | CCAGACCGCGACAGGCCAGAAC  AGTGAGGCACTGAGATGCCCCGAG |
| BRACHYURY | ATGCAGTGACTTTTTGTCGTGG  ACTGAGGCTGCATTTCCTTCTT |
| E-CADHERIN | GAGCTACACGTTCACGGTGCCC GGCTGTCCTTTGTCGACCGGTG |
